# P02 An outpatient home IV antibiotic therapy programme in a tertiary referral pulmonary infection centre: safety, cost-effectiveness, and service outcomes

**DOI:** 10.1093/jacamr/dlaf239.006

**Published:** 2026-01-05

**Authors:** Milton James Micallef, Novis Kola, Lucy Carson, Uta Hill

**Affiliations:** Royal Papworth Hospital NHS Foundation Trust, Papworth Road, Cambridge Biomedical Campus, Cambridge, CB2 0AY, UK; UNSW Sydney, Prince of Wales Clinical School, Randwick, New South Wales 2031, Australia; Royal Papworth Hospital NHS Foundation Trust, Papworth Road, Cambridge Biomedical Campus, Cambridge, CB2 0AY, UK; Royal Papworth Hospital NHS Foundation Trust, Papworth Road, Cambridge Biomedical Campus, Cambridge, CB2 0AY, UK; Royal Papworth Hospital NHS Foundation Trust, Papworth Road, Cambridge Biomedical Campus, Cambridge, CB2 0AY, UK

## Abstract

**Objectives:**

To evaluate the safety, cost-effectiveness, and service outcomes of a patient/carer-administered home IV antibiotic therapy (OPAT) programme for patients with chronic and complex pulmonary infections, and to identify best practices and areas for service optimization.

**Methods:**

A retrospective audit was conducted of all patients treated via our patient/carer-administered OPAT service between 1 January and 31 December 2023, excluding those who opted out via the NHS National Data Opt-out service (*n*=26 patients, 39 courses excluded). All eligible patients, including people with cystic fibrosis (pwCF) and those with non-cystic fibrosis-related lung disease (non-CF), were included. Data collected included demographics, patient cohort, patient experience with our model, mode of IV access (long line, peripherally inserted central catheter, totally implanted vascular access device), antibiotic regimen, duration of treatment, adverse events, reasons for early cessation, sputum microbiology, C-reactive protein (CRP) at start and end of therapy. Cost savings were calculated using NHS reference inpatient bed-day costs.

**Results:**

A total of 413 treatment episodes were delivered to 274 patients (74 pwCF courses, 339 non-CF courses). The mean treatment duration was 13·8 days (SD 4·4; range 2–32). Sputum cultures were attempted in 91% (*n*=374) of courses, with at least one isolate in 263 specimens and no significant growth in 110; only six cases lacked recent microbiology to guide therapy. *Pseudomonas aeruginosa* was the most frequent isolate. Mean CRP at start was 14 mg/L (SD 24; range 3–200), and at end 11 mg/L (SD 20; range 3–219), with a mean reduction of 4 mg/L. Seventy-three courses ended before 10 days: 25 due to side effects or allergy, 5 due to marked improvement, 1 at patient request, and 7 due to hospital admission. Reported adverse events included non-severe cutaneous (*n*=10), ill-defined/subjective (*n*=6), gastrointestinal (*n*=4), liver function derangements (*n*=3), hyponatraemia (*n*=1), and fluid overload (*n*=1). No line-related bloodstream infections occurred. Two patients tested positive for *Clostridioides difficile*, however, one of these was toxin-negative, and the other was positive 6 months after the course of home IV antibiotics. The programme saved 5682 inpatient bed-days, equating to £3·9M in cost savings (76% reduction) compared with inpatient care, exceeding published benchmarks (40%–56%).

**Conclusions:**

Home IV antibiotic treatment for complex respiratory infections, self or carer-administered in a tertiary ambulatory care setting is safe, highly cost-effective, and patient-centred. Cost savings substantially exceed published estimates. Future improvements should focus on ways to improve antibiotic stewardship, including early response-guided treatment shortening.

